# Cortical GABAergic Interneuron/Progenitor Transplantation as a Novel Therapy for Intractable Epilepsy

**DOI:** 10.3389/fncel.2018.00167

**Published:** 2018-06-26

**Authors:** Qian Zhu, Janice R. Naegele, Sangmi Chung

**Affiliations:** ^1^Translational Stem Cell Neurobiology Laboratory, Department of Cell Biology and Anatomy, New York Medical College, Valhalla, NY, United States; ^2^Hall-Atwater Laboratory, Department of Biology, Program in Neuroscience and Behavior, Wesleyan University, Middletown, CT, United States

**Keywords:** intractable epilepsy, GABAergic interneurons, cell transplantation, animal models, human pluripotent stem cells, differentiation

## Abstract

Epilepsy is a severe neurological disease affecting more than 70 million people worldwide that is characterized by unpredictable and abnormal electrical discharges resulting in recurrent seizures. Although antiepileptic drugs (AEDs) are the mainstay of epilepsy treatment for seizure control, about one third of patients with epilepsy suffer from intractable seizures that are unresponsive to AEDs. Furthermore, the patients that respond to AEDs typically experience adverse systemic side effects, underscoring the urgent need to develop new therapies that target epileptic foci rather than more systemic interventions. Neurosurgical removal of affected brain tissues or implanting neurostimulator devices are effective options only for a fraction of patients with drug-refractory seizures, so it is imperative to develop treatments that are more generally applicable and restorative in nature. Considering the abnormalities of GABAergic inhibitory interneurons in epileptic brain tissues, one strategy with considerable promise is to restore normal circuit function by transplanting GABAergic interneurons/progenitors into the seizure focus. In this review, we focus on recent studies of cortical GABAergic interneuron transplantation to treat epilepsy and discuss critical issues in moving this promising experimental therapeutic treatment into clinic.

## Introduction

Epilepsy is a serious and chronic brain disorder that affects more than 70 million people worldwide (Singh and Trevick, 2016). The fundamental characteristics of epilepsy include recurrent seizures and loss of consciousness caused by unpredictable and abnormal electrical discharges (Baraban et al., 2009; Yuan et al., 2011). The etiology of epilepsy is complicated and heterogenous, including structural, genetic, infectious, metabolic, immune and/or other unknown causes (Scheffer et al., 2017). Currently, epilepsies are classified into focal epilepsies, generalized epilepsies, combined generalized and focal epilepsies, and unknown epilepsies based on the types of seizures the patients display (Fisher, 2017; Fisher et al., 2017; Scheffer et al., 2017). In various etiologies and manifestations of epilepsies, more than 30% of cases are comprised of refractory epilepsies in which medical therapies fail to manage the seizures (Engel, 2014), presenting an urgent, unmet need for new treatments. In this review, we will discuss the promise of neuronal cell-based transplantation therapies for epilepsy as a novel and restorative therapy.

## Current Treatment for Epilepsy

Antiepileptic drugs (AEDs) are the mainstay of epilepsy treatments for controlling seizures (Goodwin, 2005) and they are cost-effective choices for most individuals with epilepsy. However, patients responding well to AEDs typically experience adverse systemic side effects, such as skin rashes, mental slowing, visual side effects, and behavioral disorders (Walia et al., 2006). For example, phenobarbital, which is one of the oldest AEDs, is associated with significant adverse events which may dramatically affect the quality of patients’ life (Baram and Shinnar, 2002). However, it is still widely prescribed because of its low cost and broad spectrum efficacy for multiple seizure types (Pal, 2007), By contrast, levetiracetam is one of the newer AEDs that has less severe side effects with higher tolerability, but its monthly cost is substantially higher (Hovinga, 2001; Ho et al., 2018). In addition, about one-third of patients that do not respond to any AEDs have to live with intractable seizures (Herman, 2010). Drug-resistant seizures can be potentially life-threatening and are associated with psychological impairments, excessive body injuries, and compromised quality of life (Sperling, 2004; Laxer et al., 2014). These disadvantages of AEDs underscore the need for alternate therapeutics that target epileptic foci.

Two alternatives are neurosurgical resection of the seizure focus (Kneen and Appleton, 2006) and neurostimulation (Lin and Wang, 2017). Compared to current drug therapies, patients that undergo surgical removal of seizure foci are more likely to become seizure free with better quality of life (Liu et al., 2018). However, surgical resection is a costly procedure, and can only be done successfully when the seizure focus can be clearly identified and safely removed without lasting consequences (Bertram, 2014; Jobst and Cascino, 2015). Moreover, many patients relapse after an initial seizure-free phase (de Tisi et al., 2011). The major advantages of neurostimulation over surgical resections and AEDs are that this approach does not require removal of brain tissue, and it can be applied into a specific target region to modulate affected neural circuity (Lin and Wang, 2017). Electrical neurostimulation is reported to be effective and acceptably safe (Heck et al., 2014), but it is currently useful only in a fraction of patients with drug-refractory seizures (Pais-Vieira et al., 2016). Transcranial magnetic stimulation is non-invasive, but there is no powerful evidence to support its efficacy (Lin and Wang, 2017). These limitations in the currently available treatment options for epilepsy, especially for those for refractory epilepsy, call for the development of more effective and restorative therapeutics.

## Cortical Gabaergic Interneurons in Normal and Epileptic Brains

In the neocortex, excitatory pyramidal cells comprise 70–80% of neurons, while interneurons, mostly inhibitory interneurons, constitute the remaining 20–30% (Markram et al., 2004). Although inhibitory interneurons releasing Gamma-Aminobutyric Acid (GABA) are far fewer than excitatory pyramidal neurons, they serve critical roles in regulating activity within brain circuits (Buzsáki et al., 2007). During early brain development, cortical GABAergic interneurons primarily arise from Medial Ganglionic Eminence (MGE) and Caudal Ganglionic Eminence (CGE), and migrate tangentially over long distances to their final destinations in the cerebral cortex where they form local inhibitory synaptic connections with excitatory pyramidal neurons and other GABAergic interneurons (Kelsom and Lu, 2013; Tyson and Anderson, 2014; Hunt and Baraban, 2015). These interneuron progenitors from MGE and CGE generate strikingly diverse subtypes of neurons, classified by their morphology, physiology and molecular properties (Markram et al., 2004).

Interneuron abnormalities have been observed in various cases of epilepsy, especially for those that were derived from MGE. In a non-human primate model of neocortical focal epilepsy, a dramatic decrease was observed in the number of GABAergic symmetric synapses in epileptic foci (Ribak et al., 1982). In tissue from patients with temporal lobe epilepsy (TLE), hippocampal seizure foci were characterized by the loss of somatostatin (SST)^+^ interneurons (de Lanerolle et al., 1989). In a rodent model of TLE, key features include: altered interneuron firing, reduced numbers of inhibitory interneurons, initial loss of inhibitory synapses followed by synaptic sprouting and alterations in GABA_A_ receptors (Sloviter, 1987; Scharfman, 1999; Kobayashi and Buckmaster, 2003; Scharfman and Myers, 2013; Chohan and Moore, 2016). With consistent observations of interneuron abnormalities in seizure foci, hypofunction/degeneration of GABAergic interneurons has been hypothesized to be key to the neural circuit dysfunction that underlies epileptogenesis and the development of recurrent spontaneous seizures (Buckmaster et al., 2002; Goldberg and Coulter, 2013; Southwell et al., 2014; Hunt and Baraban, 2015). In line with this hypothesis, it was shown that epilepsy-related loss of hilar SST^+^ interneurons resulted in reduced dentate gyrus inhibition (Hofmann et al., 2016). Furthermore, rapid optogenetic stimulation of hippocampal parvalbumin (PV)^+^ interneurons effectively suppressed spontaneous seizures (Armstrong et al., 2013; Krook-Magnuson et al., 2013), pointing to the efficacy of modulating GABAergic inhibition for therapeutic purposes.

Unlike cortical GABAergic interneurons derived from MGE, cortical GABAergic interneurons originating in the embryonic CGE, are comprised chiefly of Calretinin (CR)^+^ and Vasoactive Intestinal Polypeptide (VIP)^+^ expressing subtypes; these subtypes are not ideal for transplantation to control seizures, since they mostly target other interneurons over excitatory pyramidal neurons, and are thought to disinhibit neural circuits, which might lead to hyperexcitability (Pi et al., 2013; Hunt and Baraban, 2015; Pelkey et al., 2017).

## Gabaergic Interneuron/Progenitor Transplantation to Treat Epilepsy

In the pioneering studies, transplantation of noradrenergic neurons was reported to suppress seizures in several different models of epilepsy (Barry et al., 1987; Bengzon et al., 1990; Bortolotto et al., 1990). However, considering the preponderance of interneuron pathologies in TLE, more recent studies have focused on transplanting GABAergic interneurons/progenitors into seizure foci. Preclinical studies in animal models of epilepsy have shown the efficacy of GABAergic interneuron/progenitor transplantation in ameliorating seizures (Löscher et al., 1998; Hattiangady et al., 2008; Baraban et al., 2009; Calcagnotto et al., 2010; Handreck et al., 2012; Maisano et al., 2012; Hunt et al., 2013; Cunningham et al., 2014; Hammad et al., 2014; Henderson et al., 2014; **Table 1**). Pioneering work by Löscher et al. (1998) revealed that transplanting rat fetal GABAergic neurons into the substania nigra (SN) led to a dramatic increase in afterdischarge thresholds and significant reductions in the severity of seizures. Although this anticonvulsant effect was not long-lasting, it did not cause adverse effects (Löscher et al., 1998). In 2012, Handreck et al. observed the anticonvulsant effects of GABA-producing cells implanted bilaterally/unilaterally into the subthalamic nucleus (STN) using a pentylenetetrazole (PTZ)-induced acute seizure model in adult rats. In addition, they observed that grafting into the STN caused more pronounced anticonvulsant effects than grafting into the SN (Handreck et al., 2012). In these two studies, GABA-producing cells were placed into sites that modulate seizures rather than the seizure focus. In future studies, it will be important to compare and determine the optimal sites for cell delivery for each type of seizure, whether it is within the seizure focus or in seizure-modulating loci.

**Table 1:** A summary of GABAergic interneuron/progenitor transplantation in animal models of epilepsy.

Reference	Cell source	Cell type	Animal models of epilepsy	Location and timing of transplantation	Results of transplantation	
Baraban et al., 2009	Mouse	MGE cells from E13.5 GFP^+^ transgenic mice	*K_v_ 1.1*^-/-^ mutant mice (a spontaneous seizure model of generalized epilepsy)	Bilateral transplantation into deep layers of cortex in mouse pups (P1-P3)	• MGE progenitors generated multiple GABAergic interneuron subtypes in the brain	
					• The frequency and duration of spontaneous seizures was reduced after MGE cell grafting	
Calcagnotto et al., 2010	Mouse	MGE cells from mouse embryos	An acute epileptic seizure model induced by MES 2 months after transplantation	Bilateral transplantation into the neocortex of neonatal normal mice	• The frequency and duration of tonic seizures were reduced in the MGE group when compared to the control group	
					• The mortality rate was also significantly decreased in the MGE group, compared to the control group	
					• The grafted MGE cells migrated and differentiated into GABAergic interneurons	
Maisano et al., 2012	Mouse	Mouse embryonic stem cell-derived neural progenitors	Pilocarpine-induced TLE model in adult mice	Bilateral transplantation into the hilus of the DG of mice 2 weeks following SE	• The majority of the grafted cells differentiated into multiple subtypes of GABAergic interneurons	
					• Extensive axonal projections were formed in the brain, although marked suppression of MFS was not observed	
					• The majority of the transplanted cells exhibited electrophysiological properties, morphologies and firing patterns consistent with endogenous hippocampal interneurons	
					• The transplanted cells were functionally integrated into the host brain circuitry	
Hunt et al., 2013	Mouse	MGE cells from E13.5 GFP^+^ mouse embryos	Pilocarpine-induced TLE model in adult mice (P51)	Bilateral injection of MGE cells into the hippocampus or amygdala of adult mice (P60-76)	In the hippocampus	In the amygdala
					• MGE progenitors dispersed and differentiated into functionally integrated mature GABAergic interneurons	• MGE progenitors were present in the basal and lateral nuclei
					• The occurrence of electrographic seizures was reduced	• Seizure frequency was not altered
					• Neurobehavioral comorbidities including aggressive reaction to handling, hyperactivity and spatial learning were restored	• Only the hyperactivity deficit was reversed. Other behavior deficits were not improved
Henderson et al., 2014	Mouse	MGE cells from E 13.5 VGAT-Venus or VGAT-ChR2-EYPF transgenic embryos	Pilocarpine-induced TLE model in adult mice	Bilateral injections into the hilus of the DG or the LEC of mice 2 weeks following SE	• The DG transplant group showed significantly fewer generalized seizures than controls, based on EEG recordings and behavioral assessment.	
					• MFS in the vicinity of MGE grafts in the dorsal hippocampus was significantly reduced in the DG transplant group, compared with the media control group and the LEC transplant group	
					• MGE cells differentiated into different subtypes of GABAergic interneurons	
					• Synaptic inhibition onto granule cells was increased in the DG transplant group compared to the media control group and the dead cell transplant group	
					• Extensive inhibitory synaptic network with granule cells was established, and these new synapses were shown by optogenetics to provide functional synaptic inhibition of granule cells	
Hammad et al., 2014	Mouse	MGE cells from E12.5 GFP mice	A Stargazer mouse model of AE	Bilateral injection into the occipital cortices of P0 mice	• The frequency and duration of AE episodes were reduced and mortality was lowered after transplantation of MGE cells	
					• Despite a low yield of integration after grafting, 93 ± 4% of the integrated cells were GABAergic	
					• The defective cortical network activity was significantly altered by MGE grafting	
Löscher et al., 1998	Rat	Fetal striatal GABAergic neurons from E14 rats	A kindling model of TLE by electrical stimulation from a bipolar electrode implanted in the basolateral amygdala	Bilateral transplantation into the SN of rats after seven ADT determinations	• The implanted neurons survived up at least to the end of the experiments	
					• A significant increase in ADT was observed in the rats with GABAergic grafts in the SN, which also displayed a marked reduction in the severity of seizures	
					• This anticonvulsant effect did not relate to any adverse effects although it was not long-lasting	
Hattiangady et al., 2008	Rat	Striatal progenitors from E15 rat embryo LGE	A SE model of chronic TLE in adult rats induced by graded intraperitoneal injections of kainic acid	Bilateral transplantation of striatal progenitors into hippocampus at 4 days post-SE after treatment with FGF-2 and caspase inhibitor.	• 9–12 months after grafting, the frequency of spontaneous recurrent motor seizures was 67–89% less than epilepsy-only and sham grafting control groups	
					• Graft cell survival equivalent to 33% of transplanted cells	
					• ∼69% of the grafted cells differentiated into various subclasses of GABAergic neurons	
					• No effects on aberrant MFS.	
Handreck et al., 2012	Rat	Genetically engineered GABA-producing cell lines:	An acute seizure model by using the timed intravenous PTZ infusion seizure test.	Bilateral and unilateral transplantation into the STN of rats	• Viable transplanted cells were visible to the end of the experiments	
		(1) Immortalized GABAergic cells (M213-2O)	PTZ seizure thresholds were determined at 2–3 days before transplantation, and different time points after transplantation (10–11 days, 3 weeks, and 5 weeks).		• The anticonvulsant effects could be induced by bilateral/unilateral transplantation of GABA-producing cells into the STN, while such effects were not able to be induced by transplantation of control cells or grafting outside the STN	
		(2) M213-2O hGAD67 cells			• Grafting into STN caused more pronounced anticonvulsant effects than grafting into SNr	
Cunningham et al., 2014	Human	MGE cells derived from hESCs	Pilocarpine-induced TLE model	Bilateral transplantation of MGE cells into the hippocampus of TLE mice 2–4 weeks after SE	• The transplanted cells displaying extensive migration developed into functional GABAergic interneurons that integrated into the neural circuitry of host brain	
					• The grafted cells presented presynaptic machinery for releasing GABA to inhibit host neurons as well as postsynaptic machinery for receiving excitatory synaptic inputs from host neurons in the hippocampus	
					• The seizure frequency was significantly reduced following hESC derived-MGE cell transplantation	
					• Behavioral abnormalities such as hyperactivity, aggressiveness and cognitive deficits were alleviated after MGE cell grafting	


*ADT, afterdischarge threshold; AE, absence epilepsy; DG, dentate gyrus; E, embryonic day; FGF-2, fibroblast growth factor-2; GFP, green fluorescent protein; hGAD, human glutamic acid decarboxylase; LEC, lateral entorhinal cortex; LGE, lateral ganglionic eminence; MES, maximum electroconvulsive shock; MFS, mossy fiber sprouting; MGE, medial ganglionic eminence; P, postnatal day; PTZ, pentylenetetrazole; SE, status epilepticus; SN, substantia nigra; SNr, substantia nigra pars reticulata; STN, subthalamic nucleus; TLE, temporal lobe epilepsy.*

More studies on cell therapy for epilepsy have directly targeted the seizure focus to replenish dysfunctional or damaged GABAergic interneurons, especially for TLE models in rodents. Hattiangady et al. demonstrated that bilateral grafting of interneuron progenitors into the hippocampus reduced the frequency of spontaneous seizures on a long-term basis (Hattiangady et al., 2008). In the same line, Hunt et al. (2013) and Henderson et al. (2014) reported that spontaneous seizures could be controlled by grafting mouse fetal MGE cells into the hippocampus of TLE mice, but not the amygdala nor entorhinal cortex. By optogenetic stimulation of the transplanted MGE cells, Henderson et al. (2014) further showed that the new inhibitory synaptic connections formed onto dentate granule cells were capable of inducing strong postsynaptic inhibition, suggesting that increased synaptic inhibition from the transplants onto dentate granule cells could provide a mechanism for seizure suppression in the TLE mice. Unconventionally, Chu et al. (2004) injected that human neural stem cells intravenously in adult rats with TLE, and observed that injected stem cells migrated to the damaged hippocampus, differentiated into GABAergic interneurons, and reduced seizures, warranting further exploration of this non-invasive cell delivery method to a damaged seizure focus. In addition to fetal-derived interneuron progenitors, Maisano et al. (2012) studied the effects of transplanting GABAergic interneurons derived from mouse embryonic stem cells into the dentate gyrus of mice with pilocarpine-induced TLE, and showed that these interneurons can functionally integrate the host brain circuitry and develop mature electrophysiological firing patterns characteristic of endogenous hippocampal interneurons.

In addition to more well-defined seizure foci as in TLE, MGE progenitor cells have also been transplanted to the neocortex to prevent seizures or elevate seizure thresholds. Transplantation of mouse fetal MGE cells into the neonatal mouse neocortex was effective in reducing the frequency and duration of seizures in a genetic model of epilepsy (Baraban et al., 2009). Although the transplants did not migrate throughout the entire cortical areas affected by Kv1.1 mutation, increasing inhibition in a limited area of the developing neocortex was enough to ameliorate seizures in these mice. Transplants of mouse fetal MGE cells also reduced seizure susceptibility and spread in an acute model of epilepsy induced by maximum electroconvulsive shock (Calcagnotto et al., 2010). Additionally, in stargazer mutant mice, which show spike and wave discharges resembling absence seizures (AE), transplanting fetal mouse MGE cells into the occipital neocortex reduced AE episodes and modified defective cortical network activity (Hammad et al., 2014). Taken together, these studies of GABAregic progenitor transplantation show that multiple graft locations, in both adult and developmental models of epilepsy, can suppress seizures or raise seizure thresholds. Further work is needed to define which sites provide the most effective seizure suppression for each seizure model.

Harvesting large numbers of MGE precursors from human embryos for clinical applications is impractical and raises ethical concerns, whereas differentiation of sufficient numbers of MGE progenitor cells from human pluripotent stem cells (PSCs), including human embryonic stem cells (hESCs) and human induced pluripotent stem cells (hiPSCs), is becoming more routine in many scientific laboratories (Goulburn et al., 2011; Germain et al., 2013; Liu et al., 2013a; Maroof et al., 2013; Nicholas et al., 2013; Kim et al., 2014). After transplantation into mouse brains, hPSC-derived MGE progenitors gradually mature into functional GABAergic interneurons and become synaptically integrated into host brain circuits (Nicholas et al., 2013; Kim et al., 2014). Most importantly, transplantation of human PSC-derived MGE cells into the hippocampus was efficacious in the pilocarpine model of TLE (Cunningham et al., 2014). In this study, Cunningham et al. (2014) showed that human PSC-derived MGE progenitors migrated robustly and developed into functional GABAergic interneurons that integrated into the neural circuitry of the host brain, suppressed seizure activity, and corrected behavioral abnormalities, such as hyperactivity, aggressiveness and cognitive deficits. This study provides proof-of-concept that GABAergic interneuron progenitors derived from human PSCs could provide a valuable and unlimited cell source for cell transplantation to treat intractable seizures.

There are various benefits of cell-based therapies for epilepsy, in comparison to more traditional treatment methods. Functionally distinct subtypes of GABAergic interneurons or other types of neurons could be generated and implanted into specific regions of the host brain (Bjarkam et al., 2009), thus obviating many of the adverse systemic side effects caused by AEDs. Furthermore, because developing neurons may be able to integrate into host brain circuits, they could provide synaptic inhibition on demand in an activity-dependent manner and circumvent issues associated with taking medications regularly and maintaining constant drug dosage. A self-regulating therapeutic system of grafted interneurons, activated by host excitatory neurons that releases GABA synaptically during periods of heightened hyperexcitability (Cunningham et al., 2014) would render the need to wear devices to detect and manage seizures obsolete. While resection of the seizure focus has been used as a last-resort interventional measure for refractory epilepsy, it carries the risk of surgical damage to otherwise healthy neural tissues, along with resultant aftereffects, such as morbidity and even permanent dysfunction (Jobst and Cascino, 2015). Although high-precision stereotactic stem cell transplantation would still be associated with neurosurgical risks, it has the advantage of leaving functional neural tissue in a relatively undisturbed state, and can also be truly restorative in nature.

## Challenges and Opportunities

**FIGURE 1:**
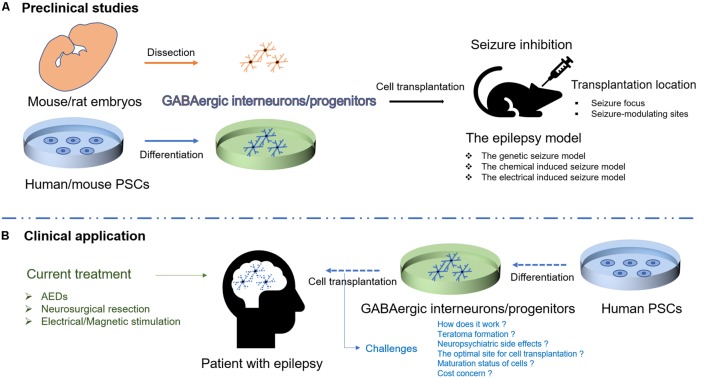
Schematic illustration of cell therapies for epilepsy. **(A)** Transplantation of GABAergic interneurons/progenitors from mouse/rat embryos or human/mouse PSCs has been studied in different rodent models of epilepsy. **(B)** Human PSC-derived GABAergic interneurons/progenitors would be a promising cell source for transplantation to target epileptic foci in humans though the challenges exist.

Transplantation of GABAergic interneurons/progenitors from mouse/rat embryos or mouse PSCs has been widely studied in the rodent models of epilepsy, and the initial results of human PSC-derived GABAergic progenitor transplantation are encouraging (Cunningham et al., 2014), offering hope for the realization of cell-based therapy for epilepsy (**Figure 1**). Nonetheless, some issues need to be addressed before transition into clinical use.

First, to better control seizures, basic mechanisms underlying seizure suppression have to be futher explored. Designer receptors exclusively activated by designer drugs (DREADDs) can be used to dissect the neural circuits responsible for different behaviors by activating or inhibiting specific neurons through modulating G protein-coupled receptor activity (Whissell et al., 2016). With this approach, the relationship between the activities of engrafted neurons and the seizure phenotype can be investigated in epilepsy models. Additionally, utilization of DREADDs could help neuroscientists determine whether seizure suppression is a result of the synaptic inhibition provided by the engrafted cells or other anticonvulsant mechanisms (e.g., graft release of neurotrophic factors including glial cell-line-derived neurotrophic factor) (Li et al., 2002; Simonato et al., 2006; Hattiangady and Shetty, 2011; Shetty, 2012).

Second, the safety of the cell preparations should be critically evaluated before transplantation. Teratoma formation is closely correlated to the persistence of proliferating or undifferentiated PSCs after transplantation (Blum and Benvenisty, 2008; Miura et al., 2009; Germain et al., 2012). Thus, for therapeutic purposes, isolating the optimal cell population before implantation will be critical for ensuring patient safety. Purifying the desired cell type by fluorescence activated cell sorting (FACS) is one approach that has been used successfully to reduce teratoma formation after transplantation (Chung et al., 2006). The ideal cell types should be highly migratory for optimal integration into host brain circuity after transplantation. In addition to the prevention of tumor formation, optimal cell density needs to be carefully determined to prevent potential adverse neuropsychiatric effects *in vivo*. None of the studies reported adverse effects of interneuron transplantation so far in the rodents. Instead, there are plethora of studies with interneuron transplantation that have shown functional benefits not only in seizure reduction but also in normalization of other behavioral abnormalities such as cognition, memory, aggressive behavior and motor symptoms in various animal models of brain disease such as epilepsy, schizophrenia and Parkinson’s (Martínez-Cerdeño et al., 2010; Liu et al., 2013b; Cunningham et al., 2014; Southwell et al., 2014; Donegan et al., 2016). Interestingly, it has been shown that the inhibition following cortical interneuron transplantation reaches a plateau with relatively low numbers of grafted cells (Southwell et al., 2014), suggesting that a larger number of cortical interneurons are not likely to cause abnormal behavior after cell transplantation. Still, the neuropsychiatric side effects need to be carefully examined in depth in the primate model that more closely matches the higher cognitive functions of the human brain. To further ensure the safety of grafts, chemogenetic modulation or suicide gene regulation may be incorporated to provide the ability to eliminate any potential adverse effects caused by the transplanted cells (Tey et al., 2007; Chen et al., 2016).

Third, the optimal site for interneuron transplantation must be determined, which is especially important when the seizure focus can not be clearly identified or there are multiple foci. As summarized above, seizure suppression has been observed following transplantation of interneurons to the seizure focus (Hunt et al., 2013; Henderson et al., 2014), but in addition, other brain regions such as SN or STN were shown to be effective sites for in modulating seizure activity in rat epilepsy models (Löscher et al., 1998; Handreck et al., 2012). Moreover, interneuron transplantation into part of epileptic brain region, providing limited innervation of the seizure focus, has also been found to reduce seizures or elevated seizure thresholds (Baraban et al., 2009; Calcagnotto et al., 2010; Hammad et al., 2014). Thus, the optimal sites of cell transplantation and distribution of the transplanted cells need to be carefully compared and determined for each seizure disorder.

Fourth, the process of maturation and differentiation in human PSC-derived MGE progenitor cells is lengthy, and appears to follow their innate *in vivo* time line in the human embryos (Nicholas et al., 2013). Developing interneurons integrate well into adult epileptic circuitry, receive excitatory inputs from the host brain, inhibit host neurons by releasing GABA and ameliorate seizures prior to attaining fully mature electrophysiological properties (e.g., Fast spiking) (Cunningham et al., 2014), but more efficient approaches need to be developed to speed up the maturation of transplanted cells with the purpose of accelerating the development of seizure suppression after grafting.

Finally, to overcome the requirement that recipients of grafts undergo long-term immunosuppression to prevent graft rejection, personalized stem cell treatments have been proposed. Personalized cell therapy uses the patient’s own iPSCs to generate needed neurons, however, this approach could easily cost hundreds of thousands of dollars (Lee et al., 2000). To overcome the prohibitive cost issue associated with personalized cell therapy, tissue banks could be established for human leukocyte antigen (HLA)-matching donor cells. This is an achievable goal for reducing costs and overcomes hazards associated with long-term immunosuppression and graft rejection (Taylor et al., 2012; Solomon et al., 2014; Cyranoski, 2017).

As the mechanisms for seizure control by interneuron grafts become better understood, and our ability to regulate the integration, proliferation, and function of engrafted cells becomes more refined, we envision a time in the future when patients with intractable seizure disorders may consider hPSC-based interneuron transplantation therapy as a safe and effective treatment option.

## Author Contributions

QZ wrote initial drafts. SC and JRN provided expert feedback and edited the manuscript.

## Conflict of Interest Statement

The authors declare that the research was conducted in the absence of any commercial or financial relationships that could be construed as a potential conflict of interest.
